# A Versatile Method to Design Stem-Loop Primer-Based Quantitative PCR Assays for Detecting Small Regulatory RNA Molecules

**DOI:** 10.1371/journal.pone.0055168

**Published:** 2013-01-31

**Authors:** Zsolt Czimmerer, Julianna Hulvely, Zoltan Simandi, Eva Varallyay, Zoltan Havelda, Erzsebet Szabo, Attila Varga, Balazs Dezso, Maria Balogh, Attila Horvath, Balint Domokos, Zsolt Torok, Laszlo Nagy, Balint L. Balint

**Affiliations:** 1 Department of Biochemistry and Molecular Biology, Research Center for Molecular Medicine, University of Debrecen Medical and Health Science Center, Debrecen, Hungary; 2 Astrid Research Ltd, Debrecen, Hungary; 3 Plant Developmental Biology Group, Agricultural Biotechnology Center, Gödöllő, Hungary; 4 Institute of Pathology, University of Debrecen Medical and Health Science Center, Debrecen, Hungary; 5 MTA-DE “Lendulet” Immunogenomics Research Group, University of Debrecen, Medical and Health Science Center, Debrecen, Hungary; 6 Center for Clinical Genomics and Personalized Medicine, Research Center for Molecular Medicine, University of Debrecen Medical and Health Science Center, Debrecen, Hungary; 7 UD-Genomed Ltd, Debrecen, Hungary; 8 Department of Computer Graphics and Image Processing, University of Debrecen Centre of Arts, Humanities and Sciences, Debrecen, Hungary; University of Connecticut Health Center, United States of America

## Abstract

Short regulatory RNA-s have been identified as key regulators of gene expression in eukaryotes. They have been involved in the regulation of both physiological and pathological processes such as embryonal development, immunoregulation and cancer. One of their relevant characteristics is their high stability, which makes them excellent candidates for use as biomarkers. Their number is constantly increasing as next generation sequencing methods reveal more and more details of their synthesis. These novel findings aim for new detection methods for the individual short regulatory RNA-s in order to be able to confirm the primary data and characterize newly identified subtypes in different biological conditions. We have developed a flexible method to design RT-qPCR assays that are very sensitive and robust. The newly designed assays were tested extensively in samples from plant, mouse and even human formalin fixed paraffin embedded tissues. Moreover, we have shown that these assays are able to quantify endogenously generated shRNA molecules. The assay design method is freely available for anyone who wishes to use a robust and flexible system for the quantitative analysis of matured regulatory RNA-s.

## Introduction

Small regulatory RNAs are short, 20-30-nucleotide-long, single-stranded, non-coding RNA molecules with various biological functions. Since the discovery in 1993 of the first small regulatory RNA, lin-4, in *Caenorhabditis elegans*, several classes of small regulatory RNA have been identified in various species. These molecules can be grouped in classes including endogenous and exogenous small interfering RNAs (siRNAs), microRNAs (miRNAs) and Piwi-interacting RNAs (piRNAs) [1]. Although the length of these small RNAs is similar, there are significant differences in their biogenesis, mode of action and biological function [1]. MiRNAs, the most extensively studied class of small RNAs, are transcribed mainly by RNA polymerase II, and processed first in the nucleus, then later in cytoplasm by endonucleases III Drosha and Dicer, respectively. Mature miRNAs are loaded into the RNA-driven silencing complex in order to act as negative regulators of gene expression by mRNA cleavage or translational repression [2]–[3]. MiRNAs are key regulators of several physiological processes in a wide range of species and experimental systems including embryonic development, cell differentiation and regulation of immune homeostasis [4]–[5]. Furthermore, these molecules play critical roles in different human diseases, including cancers, neurodegenerative disorders and autoimmune diseases [6]–[8].

Next generation sequencing initiated and expanded the identification of several new, rare or tissue-specific small RNAs in an unprecedented manner. In recent years, hundreds of new small RNA variants have been identified by next generation sequencing [9]–[12]. However, one important limitation of the current RNA sequencing approaches for studying small RNAs is the complexity of sample preparation that makes it difficult to generate quantitative data across large sample numbers [13]. Therefore, to generate quantitative data through several sample sets of newly identified or well known small RNAs, Northern-blot or quantitative PCR remain important techniques in these studies.

Stem-loop RT-qPCR was developed for specific and efficient quantification of small RNAs and became a widely used technique primarily for canonical, well-characterized small RNAs [14]. An advantage of the method is that it enables the specific detection of the mature, processed miRNA molecules even from nanograms of total RNA. This technique includes two steps: small RNA specific stem-loop primer-based reverse transcription and quantification of RT products using conventional TaqMan™ assay with a small RNA specific TaqMan™ probe and forward primer [14]. The technology is widely used for detecting well-annotated miRNA molecules from humans or common model organisms. A simplified setup of stem-loop RT-qPCR uses a stem-loop specific UPL probe (Universal ProbeLibrary) during the quantification of RT products without decreasing specificity or efficiency [15]. This method could be easily adopted into identifying and validating miRNA molecules. One limitation to widespread use of this type of qPCR assays is the lack of easy-to-use assay design software.

We developed and tested new UPL-probe based stem-loop qPCR assay design software, which proved to be adequately specific and sensitive in various applications. Excellent quantitation can be achieved in miRNA molecules that have differences as small as a single nucleotide. The designed assays were successfully tested on various types of samples, even on RNA samples isolated from formalin-fixed paraffin-embedded tissues. Moreover, we have shown that these assays can be successfully used to quantitate the level of siRNA introduced in model cell lines. Currently the transfection efficiency of siRNA molecules can be measured by microscopy or flow cytometry using labeled siRNA molecules. Our method can measure the level of the siRNA by qPCR, and this approach could replace microscopy or flow cytometry-based methods currently used. Another advantage of the approach is that the method allows for detection of the matured endogenously generated siRNA molecules from samples generated by using lentiviral transduction systems, a unique feature of the stem-loop qPCR assay system.

## Materials and Methods

### Ethics Statement

Mice: C57BL/6 J mice were sourced from Charles River Laboratories International Inc. (Germany). Animals were housed under minimal disease conditions and the experiments were carried out under institutional ethical guidelines and licenses (file number: 120/2009/DE MAB).

#### Tumor samples

Samples were collected according to the rules and regulations of the University of Debrecen, Medical and Health Science Center, with the approval of the local ethics committee (file number: RKEB/IKEB 3013–2009).

### Knock-down Mouse ES Cell Generation

shRNA lentiviral plasmid (MISSION shRNA, TRCN0000018493) was purchased from Sigma-Aldrich Ltd. for targeting the mouse PRMT1. Non-target shRNA (cat no SHC002, Sigma-Aldrich Ltd.) was used as a control. Genetically modified mouse D3 ESCs were cultured on 0.1% gelatin coated plates in feeder-free condition in 5% CO_2_ at 37°C. The ES cell medium was prepared by supplementing DMEM Glutamax media with 15% FBS, 1000U of leukemia-inhibiting factor, penicillin/streptomycin, L-glutamine, non-essential amino acids and 2-mercaptoethanol. Cells were maintained under puromycin selection.

### RNA Amplicons

Hsa-mir-181a, b, and c RNA amplicons were obtained from Sigma-Aldrich. Amplicons were diluted in a solution of 5 ng/µl yeast tRNA.

### RNA Isolation

Total RNA was isolated from ES cells and mouse organs using Trizol Reagent (Life Technologies/Invitrogen) according to the instructions. Total RNA was extracted from virus infected plants displaying symptomatic systemically infected leaves. The homogenized plant materials were resuspended in 600 µl of extraction buffer (0.1 M glycine-NaOH, pH 9.0, 100 mM NaCl, 10 mM EDTA, 2% sodium dodecyl sulfate, and 1% sodium lauroylsarcosine) and mixed with an equal volume of phenol. The aqueous phase was treated with equal volumes of phenol and chloroform, precipitated with ethanol, and resuspended in sterile water. Regarding miRNA isolation from formalin-fixed paraffin embedded (FFPE) tumours, samples were isolated from paraffin embedded cancer tissues and their adjacent non-tumorous tissues. After morphological analysis, the 5 µm thin serial sections were cut and the selected area was microscopically dissected. Samples were collected for miRNA isolation as described earlier [16]. MiRNA was isolated by using a High pure miRNA isolation kit from Roche (cat no: 05080576001) according to the manufacturer’s instructions.

### Small RNA Northern Blot Analysis

Small RNA Northern blot analysis was done, as previously described, [17] using LNA modified oligonucleotide probes. Briefly, for small RNA Northern blot analyses, the total RNA samples (8–10 µg) were fractionated on denaturing 12% polyacrylamide gels containing 8 M urea, transferred to Nytran N membrane (Schleicher & Schuell, Germany) by capillary method and fixed by ultraviolet cross-linking. Membranes were probed with 32P-labeled LNA oligonucleotide probes (Exiqon, Denmark), complementary to the mature microRNAs. 5 pmol of each oligonucleotide probe was end-labeled with [γ32P]ATP by using T4 polynucleotide kinase. Prehybridization of the filters was carried out in 50% formamide, 0.5% SDS, 5×SSPE, 5×Denhardt’s solution and 20 µg/ml sheared, denatured, salmon sperm DNA. The hybridization was carried out at 50°C for 2 hours in the same buffer. Washing of the membranes was done for 10 minutes two times with washing solution containing 0,1% SDS and 2×SSC at the temperature of the hybridization.

### Western Blot Analysis

Whole cell protein extracts were separated by denaturing SDS electrophoresis in 12.5% polyacrylamide gel and then transferred to Immobilon-P Transfer Membrane (Millipore Crp., Billerica, Massachusetts). Membranes were probed with anti-PRMT1 (07-404; Millipore Crp.) or anti-actin (A2066, Sigma-Aldrich Ltd.) antibodies, according to the manufacturer’s recommendations.

### mRNA Specific RT-qPCR

cDNA synthesis was performed with Tetro Reverse Trancriptase kit (Bioline Ltd.) according to the manufacturer’s recommendation. Quantitative PCR was performed using real-time PCR (ABI PRISM 7900, Applied Biosystems). PRMT1 (Mm0048135_g1) TaqMan® gene expression assay was used and quantified by the comparative ΔC_T_ method and normalized to GAPDH (Mm99999915_g1) expression.

### Stem-loop RT-qPCR Assay Design

Small RNA specific stem-loop RT-qPCR assays were designed using our developed UPL-probe based stem-loop quantitative PCR assay design software (http://mirnadesigntool.astridresearch.com). Matured small RNA sequences used to design the qPCR assays and the designed oligonucleotides used in this study are listed in Table 1 and Table 2. The assay design software is freely available on-line at two different locations: 1.http://mirnadesigntool.astridresearch.com. 2. http://genomics.dote.hu:8080/mirnadesigntool/The software is Open Source under the GNU GPL license and access to the source code can be requested by contacting the corresponding author.

**Table 1:** Sequences of matured small RNA molecules investigated. All sequences are in written in 5′-3′ direction.

small RNA	mature small RNA sequence
ath-mir-168	UCGCUUGGUGCAGGUCGGGAA
snoR41Y	GUUUUAUUGUCAUCUGAUUCUCAUGAUGAAUAUAUACCUCCUACUCAUUCUGAGUG
mmu-mir-1	UGGAAUGUAAAGAAGUAUGUAU
sno202	GCUGUACUGACUUGAUGAAAGUACUUUUGAACCCUUUUCCAUCUGAUG
hsa-mir-181a	AACAUUCAACGCUGUCGGUGAGU
hsa-mir-181b	AACAUUCAUUGCUGUCGGUGGGU
hsa-mir-181c	AACAUUCAACCUGUCGGUGAGU
hsa-mir-155	UUAAUGCUAAUCGUGAUAGGGGU
mmu-mir-155	UUAAUGCUAAUUGUGAUAGGGGU
RNU43	GAACUUAUUGACGGGCGGACAGAAACUGUGUGCUGAUUGUCACGUUCUGAUU
siPRMT1	TTTGGATGTCATGTCCTCAGC

**Table 2:** Sequences of oligonucleotides used for qPCR measurements. UPL probe number 21 was used for the measurements.

small RNA	stem-loop RT primer	small RNA specific forward primer
ath-mir-168	GTTGGCTCTGGTGCAGGGTCCGAGGTATTCGCACCAGAGCCAACTTCCCG	GTTTTCGCTTGGTGCAGGT
snoR41Y	GTTGGCTCTGGTGCAGGGTCCGAGGTATTCGCACCAGAGCCAACCACTCA	GTTGTTTTATTGTCATCTGATTCTC
mmu-mir-1	GTTGGCTCTGGTGCAGGGTCCGAGGTATTCGCACCAGAGCCAACATACAT	GTTGGGTGGAATGTAAAGAAGT
sno202	GTTGGCTCTGGTGCAGGGTCCGAGGTATTCGCACCAGAGCCAACCATCAG	GTGCTGTACTGACTTGATGAAA
hsa-mir-181a	GTTGGCTCTGGTGCAGGGTCCGAGGTATTCGCACCAGAGCCAACACTCAC	GGAACATTCAACGCTGTCG
hsa-mir-181b	GTTGGCTCTGGTGCAGGGTCCGAGGTATTCGCACCAGAGCCAACACCCAC	GTTTGAACATTCATTGCTGTCG
hsa-mir-181c	GTTGGCTCTGGTGCAGGGTCCGAGGTATTCGCACCAGAGCCAACACTCAC	GGGAACATTCAACCTGTCG
hsa-mir-155	GTTGGCTCTGGTGCAGGGTCCGAGGTATTCGCACCAGAGCCAACACCCCT	GTGGGTTAATGCTAATCGTGAT
mmu-mir-155	GTTGGCTCTGGTGCAGGGTCCGAGGTATTCGCACCAGAGCCAACACCCCT	GGGGGTTAATGCTAATTGTGAT
RNU43	GTTGGCTCTGGTGCAGGGTCCGAGGTATTCGCACCAGAGCCAACAATCAG	GTGAACTTATTGACGGGCG
siPRMT1	GTTGGCTCTGGTGCAGGGTCCGAGGTATTCGCACCAGAGCCAACGCTGAG	GGGGTTTGGATGTCATGTC

Universal reverse primer in all cases was: GTGCAGGGTCCGAGGT. All sequences are in written in 5′-3′ direction.

A public miRNA assay sequence depository has been initiated at http://genomics.med.unideb.hu/Research_Interest/miRNA_Research and a professional support group has been initiated at Linkedin entitled, “miRNA detecting QPCR assays”.

### Small RNA Specific Stem-loop RT-qPCR

Small RNAs were transcribed into cDNA via miRNA specific reverse transcription reaction using the designed miRNA specific stem loop-RT primer and Transcriptor First Strand cDNA Synthesis Kit (Roche). Reverse transcription reaction was prepared according to manufacture’s instructions with modifications. Initial amount of RNA was 10 ng per reaction while applied primer was miRNA specific stem-loop primer in 50 nM final concentration. Small RNA quantification was performed by quantitative real-time RT-PCR using Light Cycler 480 Master (Roche), designed universal reverse primer, and miRNA specific forward primer, as well as Universal Probe Library probe 21 (Roche). All measurements were done in triplicates on a LC480 qPCR instrument.

### miRNA Specific Stem-loop RT-qPCR (Life Sciences)

Small RNAs were transcribed into cDNA via miRNA specific reverse transcription reaction using miRNA specific stem loop-RT primer and TaqMan® MicroRNA® Reverse Transcription Kit (Life Sciences). Small RNA quantification was performed by quantitative real-time (reverse transcriptase) PCR (polymerase chain reaction) using Taqman Gene Expression Assays (Life Technologies). The following TaqMan® gene expression assays were used in small RNA quantification: mmu-mir-155 (002571) and snoRNA 202 (001232).

## Results

Guegler and colleagues described the original stem-loop based miRNA quantitation method in 2005 [14]. Benefits of this method include high specificity and increased sensitivity compared to classical methods such as Northern blot, while maintaining the ability to distinguish between mature miRNAs and their precursors. While early discoveries based on cloning of miRNA molecules assumed that the number of miRNAs in a particular sample is in the range of hundreds, recent work based on next generation sequencing methods showed that the number of miRNA variants in a cell is increased by orders of magnitudes. Various processes that affect both 5′ and 3′ ends of these molecules increase the complexity of the miRNA world. While a couple of hundred small regulatory RNA molecules could be detected with a library of PCR based assays, these novel findings aim for a more flexible detection method that can be used for validation or for further studies of the previously identified molecules. Another important aim of our research was to expand the number of available tools to discover small regulatory RNA molecules in non-canonical or less characterized model systems.

Our goal was to develop and test a robust and flexible stem-loop primer-based qPCR assay design algorithm that can be used in a wide range of applications using mouse, human cell lines, tissues, plants and pathological samples. Furthermore, we decided to introduce the qPCR-based small RNA detection assay into the quantitation of transduced shRNA derived mature siRNA molecules that are commonly used in biological research.

**Figure 1:**
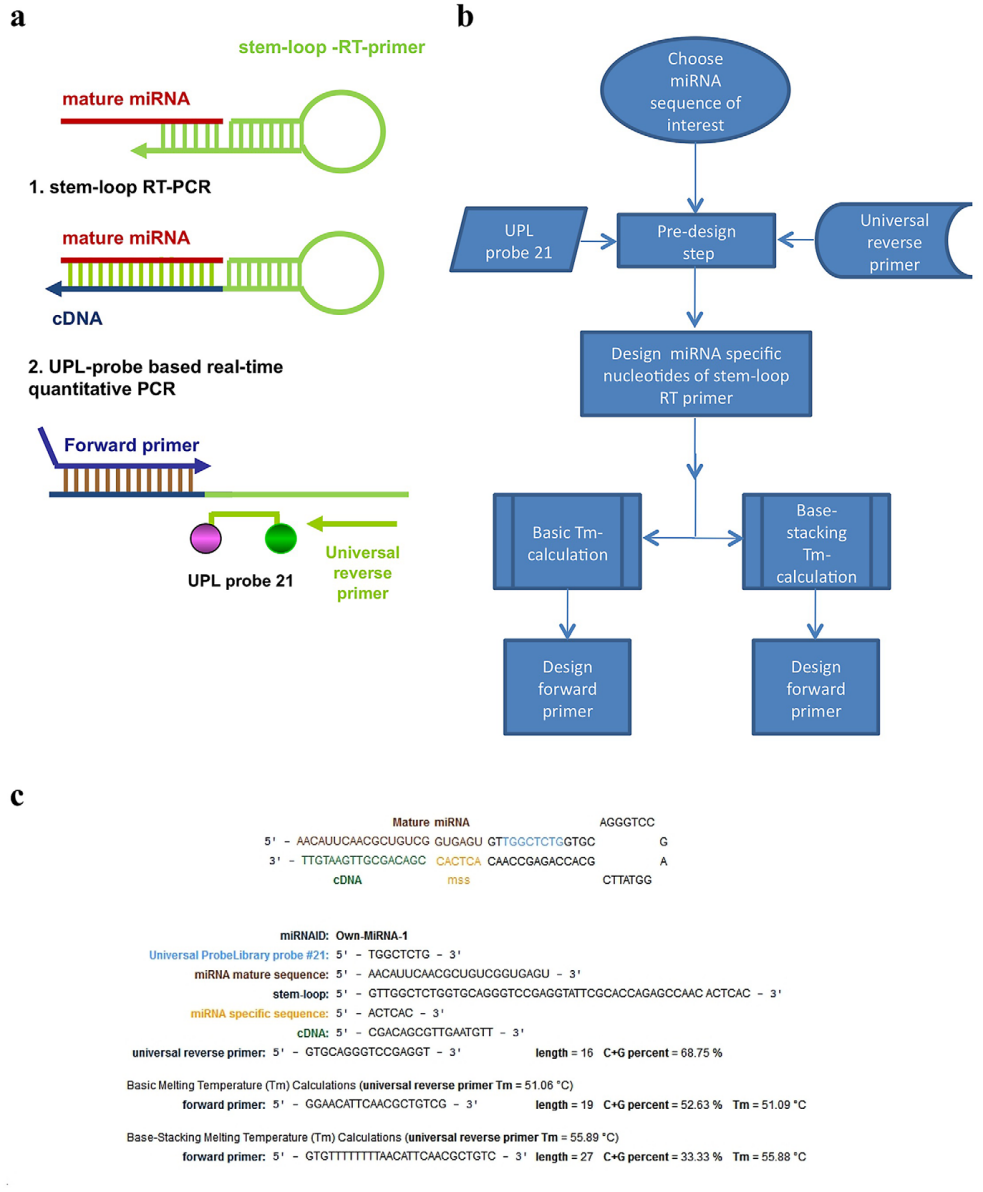
Schematic description of small RNA specific UPL-based quantitative PCR assay and our oligo design system. Two steps small RNA specific UPL-based quantitative PCR assay relies on reverse transcription using small RNA specific stem-loop RT primer and real-time quantitative PCR reaction using small RNA specific forward primer, UPL21 probe and universal reverse primer (A). Workflow of our oligo design system (B). Primers and probe for the designed hsa-mir-181a specific UPL-based quantitative PCR assay (C).

The development of our primer design system was based on the original publication [15], using the concept described earlier [14] and by replacing the miRNA specific unique TaqMan™ probe with an LNA (locked nucleic acid) probe that is specific for the hairpin oligonucleotide. Other components that provide specificity for the system were maintained, namely: sequence specific stem loop RT primer and miRNA specific forward primer (Figure 1A).

Assay design starts with the introduction of virtually any small RNA molecule sequence and follows the workflow described in Figure 1B. On a separate page, under the “Other Tools” menu, the software allows searching for groups of miRNA-s based on their identifiers, on their position relative to annotated genes or Gene Ontology groups. The output of the design algorithm is as follows: (1) the stem-loop oligo for the reverse transcription step, (2) the universal reverse primer, (3) the sequence specific forward primer, where (2) and (3) are needed for the amplification step of the reaction i.e. the qPCR quantification. The results page of the oligo design software highlights the miRNA specific sequence of the stem loop primer, the melting temperature (Tm) of the designed oligos, with two different calculation methods. The system uses a commercially available LNA modified probe, UPL 21 (Universal Probe Library 21) (Figure 1C).

**Figure 2:**
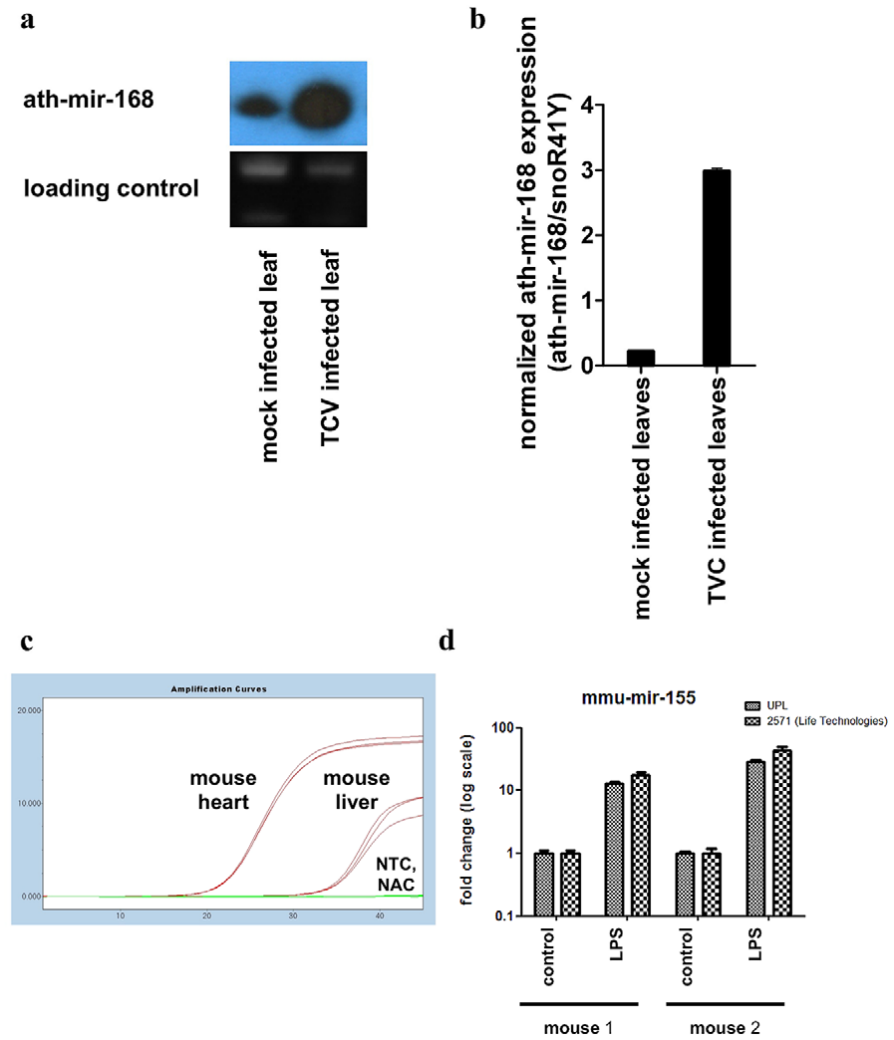
Validation of miRNA specific UPL-based quantitative PCR assays. Arabidopsis thaliana specific miR168 expression in TCV infected leaves compared to mock control investigated by Northern-blot. As normalizer we used ethidium bromide staining of ribosomal RNA (A) Same samples as in panel “A” were measured with miRNA specific UPL-based quantitative PCR assays. As normalizer we used snoR41Y specific assays. (B). Quantification of LPS induced mir-155 level in mouse macrophages by both UPL-based qPCR system and mature miRNA specific qPCR assays of Life Technologies. As normalizer we used sno202 specific assays. (C).

In order to test if the system gives comparable results with the most widely accepted miRNA detection method, namely Northern blot, we compared the expression of miR-168 in turnip crinkle virus (TCV) infected or mock transfected leaves of *Arabidopsis thaliana*. We have previously shown that miR-168 is induced during viral infections [18]. In Figure 2A we provide data that TCV infected leaves have a high level of miR-168 compared to mock infected leaves. Based on our qPCR measurements from the same samples, the normalized expression level of miR-168, relative to snoR-41Y, was higher in the infected leaves compared to the mock infected ones similar to the results observed by Northern blot, as seen in Figure 2 panel A and panel B. It has to be noted that the sensitivity of the qPCR measurement is higher than the sensitivity of the Northern blot measurement. The input material for a Northern blot is in the range of micrograms while a successful qPCR can be performed from as little as 10 nanograms total RNA. On the other hand qPCR is not giving any information about the size of the miRNA molecule. These results show that the qPCR assays allow a specific and sensitive quantification of target molecules in plant systems and in some specific applications when input material is limited, qPCR based assays can replace Northern blot analysis.

To further test the reliability of this modified stem-loop-based qPCR system, we investigated the well characterized tissue specific miRNA, mir-1, which was shown previously to be expressed at high levels in the heart [19]. Based on the publicly available data in the www.microrna.org database, in liver tissue, mir-1 has a low expression level. We isolated total RNA from the previously mentioned mouse tissue and as shown in Figure 2C, we could detect a substantial difference between the samples derived from the specified tissues with a difference of 13 Cp, which corresponds to more than three orders of magnitude difference in expression levels. In all measurements, the control lacking the reverse transcriptase enzyme (NAC: no amplification control) and the control lacking the template (NTC: no template control) produced no amplification curves. Samples were normalized to sno-202, which gave similar Cp values across the two types of tissues (data not shown).

Comparing the performance of our assay to other assays based on similar principles was an important step of our investigation. The stem-loop RT primer assays are one of the most reliable miRNA quantitation methods due to their ability to distinguish the matured miRNA molecules from their precursors. One of the other assay available on the market is using probes that are designed on the junction of the matured miRNA with the stem-loop RT primer. The system used by us is simplifying this approach by using a universal probe that binds to the stem-loop RT primer. We decided to measure the induction of mir-155 upon LPS treatment in mouse bone marrow derived macrophages with both assay systems. Our results presented in Figure 2 D show that both assays systems are able to detect the induction of mir-155 upon LPS treatment in a similar manner in two biological replicate samples.

**Figure 3:**
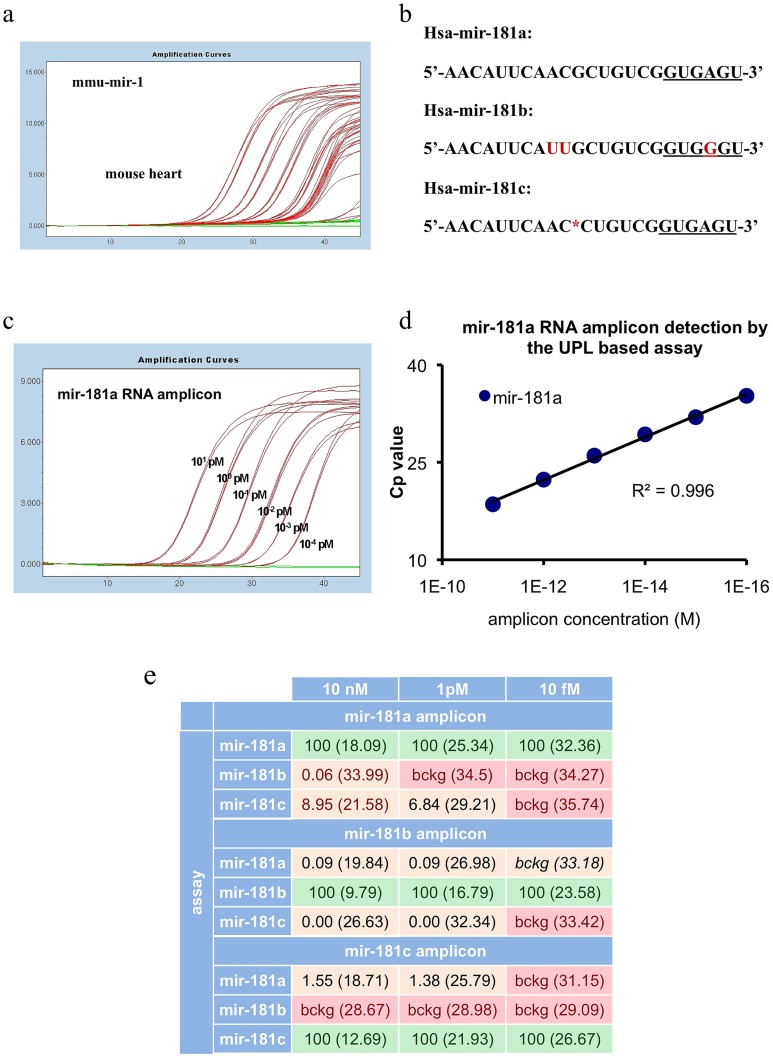
Sensitivity and specificity of miRNA specific UPL-based quantitative PCR system. Amplification plot of mmu-mir-1 in range from 10 ng to 10^–^3 ng input mouse heart total RNA (A). Sequence similarities and differences between mir-181a, b, and c (B). Amplification plot of synthetic mir-181a miRNA ranging from 10 pM to 10^–4^ pM input mir-181a amplicon (C). Standard curve of synthetic mir-181a miRNA (D). Specificity and relative detection capacity of mir-181 specific UPL-based qPCR assays. Numbers represent the percentage of the signals measured on the synthetic amplicons. 100% is always the signal measured by an assay on its specific synthetic amplicon, like mir-180a assay on the mir-181a synthetic amplicon. In brackets the corresponding Cp values are shown.(E).

In order to test whether we can detect as little as two fold differences across a wide dynamic range of sample concentration, we generated dilution curves from 10 nanograms to 100 femtograms of mouse heart total RNA, and each point in the dilution curve was further diluted two fold. As shown in Figure 3A, we could measure two-fold differences down to 1 picogram, but the presence of the target was detected even at the range of 100 femtograms of total RNA per reaction. At such low concentrations, the assay could detect the presence of the template, but not the two-fold differences probably due to stochastic events in the reaction.

A very important issue in the field of small regulatory RNA molecules is the ability to distinguish between miRNAs that have tiny differences in their sequence. We selected the family of mir-181 miRNAs that has several members, and we compared mir-181a, mir-181b and mir-181c. As shown in Figure 3B, mir-181b differs from mir-181a by three nucleotides: one in the binding region of the stem loop primer and two internal nucleotides, while hsa-mir-181c is separated from hsa-mir-181a by a single missing internal nucleotide. Figure 3C shows a dilution curve of chemically synthesized RNA template ranging from 10 picomoles down to 100 attomoles. As shown in Figure 3D, in this range, the assay generated for the detection of mir-181a was able to detect the targets in a linear manner across a dynamic range of six orders of magnitude. The assays generated to detect the other two members of the family had a similar performance (data not shown).

We investigated the specificity of the assay system by testing if the qPCR measurement gives any non-specific signal on another synthetic RNA molecule from the mir-181 family. The specific assays for the three members of the family namely mir-181a, mir-181b and mir-181c were used on the mir-181a synthetic RNA amplicon in the following amplicon concentrations: 10 femtomoles, 1 picomole and 10 nanomoles. In parallel measurements, the three assays were tested on the amplicons of two other members of the mir-181 family namely mir-181b and mir-181c. Results are presented in Figure 3E. Measurement on mir-181a amplicon in the concentration of 10 nanomoles and 1 picomole, with the two non target assays, showed a 0.06% and background non specific signal for mir-181b and 8.95% and 6.84% for mir-181c at 10 nanomoles and 1 picomole concentrations respectively compared to the signals given by the mir-181a specific assay. At 10 femtomoles concentration of the mir-181a amplicon the two non target assays for mir-181b and mir-181c gave only a background signal. It has to be noted that the background signal was not changing with the concentration of the non specific template. Similar results were seen with the two other amplicons, namely mir-181b and mir-181c. For details see Figure 3E.

**Figure 4:**
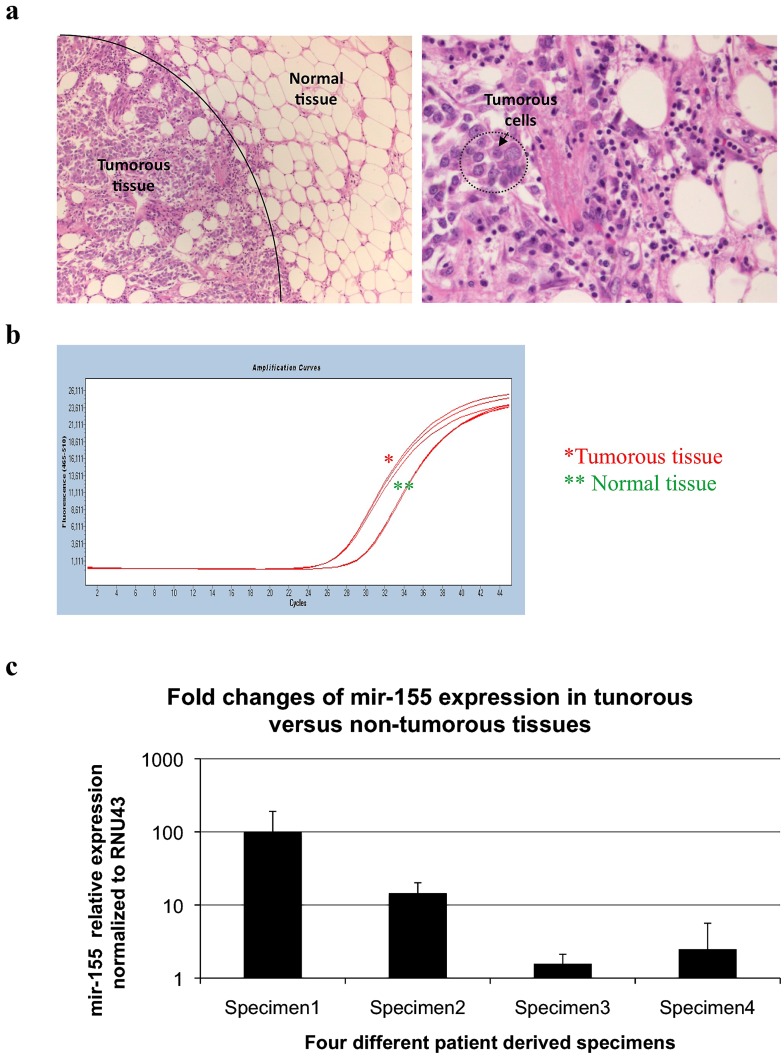
Quantification of miRNA expression from FFPE samples. Cancerous tissue and surrounding normal tissue obtained from microdissected human FFPE breast cancer sample [invasive duct carcinoma]. Images are showing hematoxylin-eosin stained sections with 10× and 40× original magnifications, respectively (A). Amplification plot of hsa-mir-155 in tumor tissue and normal tissue (B). Fold expression of RNU43 normalized hsa-mir-155 expression in tumor tissues relative to surrounding non-tumorous tissues from four independent breast cancer derived specimens. Error bars represent the standard deviations of the triplicate qPCR measurements (C).

One important application for miRNAs is as biomarkers. Several miRNAs were shown to have different expressions in tumor tissues. The importance of miRNAs as biomarkers is based on their stability and the possibility of detecting them from formalin-fixed paraffin-embedded samples. We generated assays against human mir-155, a miRNA reported to have differential expression levels in mammalian cancers [20]–[21]. We separated tumor and normal tissues by using microdissection (Figure 4A) and RNA was isolated as described in the Materials and Methods section. As shown in Figure 4B, samples of FFPE origin produced good amplification curves. The increased expression levels of mir-155 in cancerous tissues compared to surrounding non-tumorous tissues, normalized to RNU43 from four independent specimens, are shown in Figure 4C. Similar tests were performed for mir-145 in breast cancer and mir-21 in colorectal carcinoma samples (data not shown).

**Figure 5:**
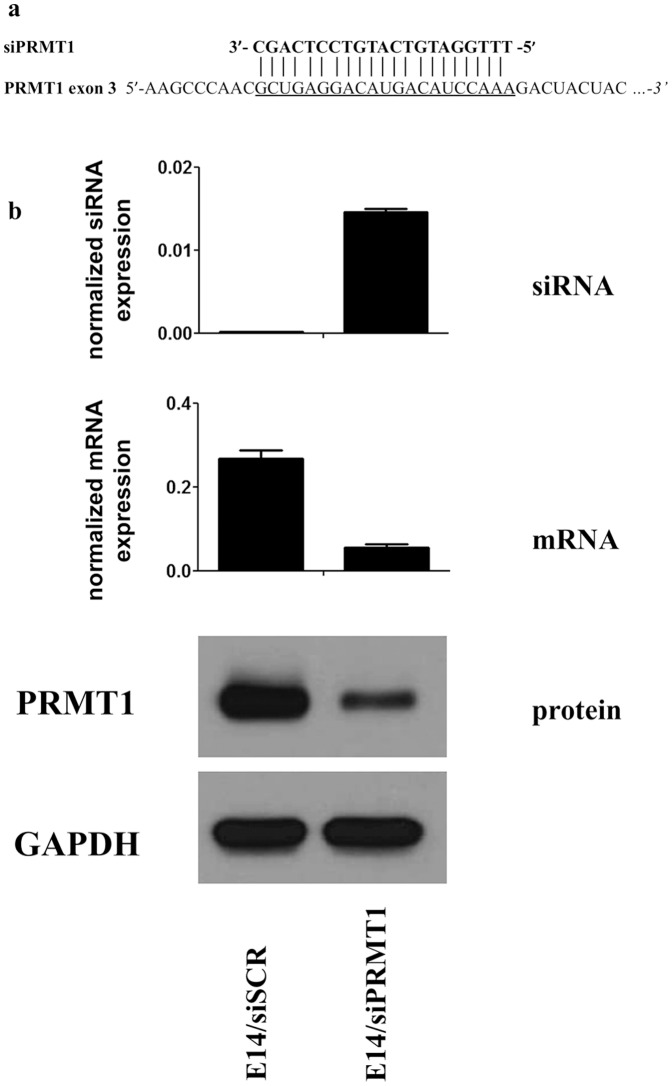
Measurement of siRNA expression with small RNA specific UPL-based quantitative PCR assays. Sequence specificity of PRMT1 specific siRNA for exon 3 of PRMT1 (A). PRMT1 specific siRNA levels as detected by qPCR and the corresponding mPRMT1 mRNA as well as protein levels detected by qPCR and Western blot analysis (B).

To test whether this system can be used to detect the commonly used siRNA molecules generated from a stably integrated plasmid system, we generated an assay that recognizes the processed siRNA targeting mouse PRMT1. The targeted sequence from the 3-th exon of mouse PRMT1 gene and its targeting siRNA are shown in Figure 5A. Cell lysates from PRMT1 silenced mouse embryonic stem cells were investigated by qPCR for measuring matured mPRMT1-specific siRNA molecules (normalized to sno202). As a negative control scrambled siRNA expressing mouse embryonic stem cells were used. In order to test the efficiency of the endogenously expressed siRNA molecules we measured by RT-qPCR the mRNA level of the target gene (normalized to GAPDH) and as an ultimate test the protein level of PRMT1 by Western Blot (loading control was actin). Results of the measurements are presented in Figure 5B. The endogenously expressed siRNA can be detected with our assays and these siRNA molecules are functionally active as shown by RT-qPCR and Western Blot. Our results show that our oligo design system can be used to generate assays that can quantify one of the most frequently used gene silencing tools: the intracellular, processed and fully active siRNA molecules.

## Discussion

Recent developments in the field of miRNA research emphasize the importance of this class of molecules. The stability of these molecules allows their detection from archived pathological samples. As biomarkers they can be detected from serum, urine and sputum [22]–[24]. New technologies used for their characterization have shown the complexity of these molecules and the importance of the validation by independent methods and from larger sample numbers. We have developed an algorithm for the design of miRNA detecting qPCR assays and generated a free, web-based software tool for the scientific community. Our tests have demonstrated that these assays are cost-effective, flexible and can be used in routine qPCR measurements from various sample types, including formalin fixed paraffin embedded tissue samples. Moreover, we have developed a new application of qPCR-based small RNA measurements, i.e. the quantification of intracellular siRNA molecules. Sharing of tools and reagents has always been a distinctive mark of the scientific community compared to other fields of society. We offer this tool to the broadest scientific community in order to expedite the validation of newly discovered miRNA molecules, to researchers working with non-canonical model organisms, and to those needing a flexible and easy to use miRNA detection method.
